# Practical considerations to guide development of access controls and decision support for genetic information in electronic medical records

**DOI:** 10.1186/1472-6963-11-294

**Published:** 2011-11-02

**Authors:** Diana C Darcy, Eleanor T Lewis, Kelly E Ormond, David J Clark, Jodie A Trafton

**Affiliations:** 1 South Bay Regional Genetics Center, Santa Clara Valley Medical Center, San Jose California, USA; 2 Program Evaluation and Resource Center and Center for Health Care Evaluation, Department of Veterans Affairs, Menlo Park California, USA; 3 Human Genetics and Genetic Counseling Program, Stanford University, Stanford California, USA; 4 Anesthesiology Department, Stanford University and VA Palo Alto Health Care System, Palo Alto California, USA

**Keywords:** genetic tests, electronic medical records, privacy, storage of genetic information, access to genetic information, types of genetic information

## Abstract

**Background:**

Genetic testing is increasingly used as a tool throughout the health care system. In 2011 the number of clinically available genetic tests is approaching 2,000, and wide variation exists between these tests in their sensitivity, specificity, and clinical implications, as well as the potential for discrimination based on the results.

**Discussion:**

As health care systems increasingly implement electronic medical record systems (EMRs) they must carefully consider how to use information from this wide spectrum of genetic tests, with whom to share information, and how to provide decision support for clinicians to properly interpret the information. Although some characteristics of genetic tests overlap with other medical test results, there are reasons to make genetic test results widely available to health care providers and counterbalancing reasons to restrict access to these test results to honor patient preferences, and avoid distracting or confusing clinicians with irrelevant but complex information. Electronic medical records can facilitate and provide reasonable restrictions on access to genetic test results and deliver education and decision support tools to guide appropriate interpretation and use.

**Summary:**

This paper will serve to review some of the key characteristics of genetic tests as they relate to design of access control and decision support of genetic test information in the EMR, emphasizing the clear need for health information technology (HIT) to be part of optimal implementation of genetic medicine, and the importance of understanding key characteristics of genetic tests when designing HIT applications.

## Background

A strength of electronic medical record (EMR) systems is that they not only provide a mechanism to store and organize patient health information but also provide the ability to filter health information based on clinical utility or relevance to individual clinical specialties, provide education and clinical decision support, and implement patient preferences around access to health information. For example, design of EMR interfaces requires decisions regarding what to present when to clinicians, as user interface design determines what information clinicians will access and notice when delivering clinical care. Genetic medicine is an area in which these strengths of EMRs are of particular importance for ensuring efficient, effective, and ethical use of what can often be complex and sensitive findings. In this manuscript we explore key factors to consider in EMR design decisions regarding access controls on genetic testing results, and discuss characteristics of classes of genetic tests relevant to how EMRs use, display, and offer decision support for genetic testing information.

Genetic testing is an increasingly common tool used throughout health care, but little has been done to optimize use of genetic information in EMRs. The number of clinically available tests is rapidly increasing, and healthcare professionals are using them on a more regular basis. At the time of this writing, tests for almost 2,000 clinical conditions exist [1]. For example, genetic tests on newborns with visible birth defects or on children with intellectual disabilities are now typically used to establish a diagnosis, which in turn can assist in determining prognosis, finding the most effective treatments, and making other health care decisions. Many patients with earlier than typical cancers (before age 50) or with extensive family histories of cancer [2] are now referred for genetic testing to help determine the most appropriate screening and prevention therapies. Genetic testing may also detect the existence of treatable metabolic disorders. These are only three examples of the current clinical uses of genetic testing. However, a recent study [3] reported that most surveyed health care professionals reported that EMRs had poor systems for online test ordering, little decision support for genetic test ordering, and treated the genetic test results similarly to other medical test results [3].

While genetic information has similarities to non-genetic personal health information there are some key differences, well-outlined in McGuire et al [4]. These distinctive properties include the uniqueness of genetic information, leading to identifiability of an individual; the predictive capability of genetic test results; the permanence of genetic changes; the impact on family members; the temporality of the data interpretation; and the history of eugenics [4]. As with many other medical tests, there are also issues that may significantly influence result interpretation (e.g. the variable and rapidly changing test sensitivity, specificity and positive predictive value, and the off-label use of genetic tests), and may therefore require significant educational support as the volume of genetic tests increases exponentially.

## Discussion

### Dimensions of genetic test results relevant to EMR systems

#### A. Characteristics of the health condition

How to display and protect genetic information in ways that allow clinicians to use this information to help patients while also protecting their privacy has been discussed and debated for years. Some genetic test results are predictive and confirm a propensity for or certainty of developing a condition prior to actually developing it (e.g. Huntington's disease, various cancers). Some of these conditions may have screening or preventative actions that are available to decrease the likelihood of disease, while others may have only psychological implications from knowing. Individuals may want personal control over access to genetic test results because they are fearful that knowledge of this information will lead to discrimination, for example in employment or insurance. They may be reluctant to be tested at all for some conditions, and if tested, reluctant to have results documented in their medical record. While few instances of pre-symptomatic genetic discrimination have been documented, the fear of such discrimination led to the federal implementation of the Genetic Information Nondiscrimination Act (GINA) of 2008 [5,6] and other state laws that provide specific genetic privacy protections.

For sensitive conditions such as certain cancers or dementias, patients may not wish others to know about their condition or their risk of developing the condition; these concerns are not different from the issues that arise with other potentially stigmatizing medical information (such as sexually transmitted diseases, HIV, mental health conditions, and learning disabilities). Patient concerns about being stigmatized based on a genetic test result will likely vary from person to person and from culture to culture. When people fear being stigmatized they will want more complete control over disclosure of genetic tests results, even to other health care providers, and may want to be consulted every time this potentially stigmatizing information is requested.

Balancing the many benefits of making genetic test results widely accessible in a patient's medical record are the potential benefits of restricting access to certain genetic test results and the need for appropriate and personalized privacy protection [see also 7-8]. While the issue of protection of genetic information specifically remains widely discussed [e.g. 4], recent trends suggest that genetic information should be accorded the same privacy protections as other health information rather than requiring special protections [9,10]. One study suggests that patients do not generally view genetic information as different from other types of medical information, but rather they make distinctions about the sensitivity of the condition being tested and who should have access to the information [11]. This study also suggested that patients feel all health information should be protected and that personal control over who accesses information was important. In an EMR patients could potentially opt for genetic test results to be safeguarded, for example with special privacy safeguards including password protected sections or a "break the glass" mechanism, where an authorized user could override access restrictions only under emergency situations [12]. Health IT systems will need to develop methods to implement these safeguards, and track patient concerns and preferences to determine when to turn on these protections.

#### B. Patient preferences to know or not know genetic test results

One of the difficulties of designing access controls for genetic test results is that different patients may react to the same test result differently, particularly when a genetic test result for a chronic condition (e.g. diabetes) or preventable condition (e.g. lung cancer, skin cancer) is deterministic based on a patient's genetic makeup [13] and when inconsistency in health care provider understanding of these test results may lead to patient confusion. For example, sometimes knowing that a condition has a genetic component may actually remove stigma and increase the likelihood that the patient and their family are proactive about managing it. Patients in this example who are told that their genetic background makes them more susceptible to diabetes may stop blaming themselves for the disease. They could adopt the analogy of myopia, and consider diet and lifestyle changes the same as wearing glasses: a logical step to address a condition they cannot change. Dieticians and other providers seeing this information in their medical record can be proactive about helping patients take positive steps to improve their - and their families' - overall health. Alternatively, other patients given the same information could become fatalistic and believe that no matter what actions they might take they are certain to develop the disease because they have a genetic predisposition. Restricting access to the information can prevent multiple health care providers from giving the patient conflicting information about their likelihood of actually developing the disease and the benefits of preventive action. In addition to reacting to test results differently, patients may be ambivalent about knowing genetic information at all. Rarely, patients may change their minds about knowing all or some of the results of a genetic test after having the test performed. An example might be a patient at risk for a late onset neurological disorder such as Huntington's disease or Amyotrophic Lateral Sclerosis (ALS).

More frequently, multiplex tests that assess a broad range of conditions in a single test can unexpectedly reveal secondary information. These tests cover more conditions than necessary to reduce the costs of test development and implementation while improving diagnostic coverage. For instance, a chromosome microarray done as a diagnostic test in a child with an intellectual disability may also identify an increased risk of adult-onset cancer when a relevant gene is identified as deleted. The ease of information storage in an EMR may mean that more information than is strictly medically necessary may then be placed in a patient's record. If a wide range of genetic information is stored in an EMR, including results from multiplex tests, some of this information will not be immediately relevant to the patient's health care. And as more and more information from genetic tests is stored in an EMR, the likelihood increases that a health care system will access and use this information in ways that improve patient care but that are unintentionally upsetting to a patient. For example, a patient could be preemptively denied access to a medication that is likely to be ineffective for them based on their genetic makeup rather than presented with choices and given the opportunity to weigh the risks and benefits.

There are serious ethical considerations that go along with inadvertently coming into possession of information about a patient's probability of developing a disease or reacting to a medication if the patient did not agree to that specific testing. Multiplex tests, such as a genome-wide single nucleotide variants (SNVs; also known as single nucleotide polymorphisms or SNPs) genotyping assessment or whole genome/exome sequencing, may be the only choice or the best choice a provider has when ordering a genetic test for a patient. A patient who underwent genotyping for one reason may also learn, for example, that she is at high risk of a serious side effect if given a specific medication that her primary care doctor would otherwise have prescribed. If the full genotyping results were stored and updated in an EMR the patient could be protected from receiving such a medication and suffering the side effects. There may also be a strong financial argument if not moral obligation to prevent patients from receiving expensive and potentially harmful drugs if it is unlikely the patients would benefit from them.

An EMR could have safeguards built in to protect against accidental revelation of unwanted information from the results of a multiplex test that are stored in the patient's medical record. One system that could be considered to address these ongoing issues would be to use the EMR or a patient interface that contacted patients on a regular basis to inquire about their preferences for privacy and information sharing. While informed consent educational materials would need to be created to support this, it would allow us to use technology interfaces to regularly prompt patients for current preferences and permissions for use of their genetic information in their health care.

#### C. Clinician characteristics and training

A genetic test can serve as a clinical diagnosis that allows for prognostication and as a specific treatment plan. For example, a woman who carries specific mutations in the *BRCA1 *or *BRCA2 *(BReast CAncer) genes has a much higher risk for breast and ovarian cancer over her lifetime than the general population. Medical recommendations for this patient will likely include more frequent screenings and the consideration of prophylactic removal of the ovaries by age 40; the patient would also be able to consider a prophylactic mastectomy [14]. It would be important in this case that her gynecologist, oncologist, and primary care doctor be aware of this test result and its associated implications. Similarly, a patient with a genetic mutation in one of the genes responsible for Lynch syndrome has a much higher likelihood of developing colorectal cancer over his or her lifetime than the general population. It would be recommended that this patient have regular colonoscopies at a much earlier age than is typically recommended [15]. It would be important for this patient's primary care doctor, gastroenterologist, and oncologist to be aware of this genetic test result and diagnosis and its implications.

While genetic tests are not the only laboratory tests or diagnostic tests that are complex and require specialized training to interpret, and misinterpretation of test results can occur in other circumstances, many health care professionals self-identify as ill-prepared and under-educated about genetic tests and how to interpret genetic test results [16,17]. Misinterpretation of any medical test can have important consequences: unintentional distress, unjustified decisions about future actions, or unnecessary current actions. Given the difficulty of interpreting genetic testing results/reports and the potentially devastating effects of misinterpretation (such as the decision to terminate a pregnancy or undergo invasive screening or even prophylactic surgery), it might be argued that genetic test results should only be provided to health professionals with specific training in genetic medicine. Alternatively, interpretation by a credentialed expert in the field could be a required part of the reporting of a genetic testing result. When deemed appropriate, an EMR could restrict access to genetic test results that have not been interpreted by an expert, or restrict access to all results (or to other tests that are complex to interpret) unless specific trainings and competencies have been demonstrated by the accessing clinician.

### Characteristics of genetic test results relevant to access control and decision support system design

The factors described above that are relevant to how EMR systems present genetic testing results are arguably not unique to genetic tests. However, as health care systems adopt electronic medical records (EMRs) that facilitate the storage of extensive genetic data, policies on access to and use of genetic test results will need to take the distinctive characteristics of genetic tests into account. EMRs offer the opportunity to take advantage of advanced access controls and decision support capabilities. To assess the technosocial needs that arise when considering how best to use EMRs to control access and use of genetic test results, we will review the types of genetic tests that are currently performed and the variation in their interpretability and complexity. How EMRs handle different types of genetic information and genetic test results will likely vary based on these characteristics. Much of this content draws on information broadly accepted in the genetics literature. Resources that provide more in-depth background on genetic conditions and clinically available testing include: the Gene Tests Web site http://www.geneclinics.org or http://www.genetests.org; OMIM: Online Mendelian Inheritance in Man http://www.ncbi.nlm.nih.gov/omim//; Genetics Home Reference http://ghr.nlm.nih.gov/; and others described in Uhlmann and Guttmacher [18].

#### A. Why all genetic tests are not created equal: Influence of test purpose on access and decision support needs

Characteristics of different types of genetic tests may have different implications for how to store the test results in the medical record and how accessible to make these results. This section reviews three main types of genetic testing: (1) testing to make a diagnosis, with the goal of improving medical treatment, (2) testing to assist in reproductive choices, and (3) testing, usually in a presymptomatic manner, to predict future health outcomes. The reasons a test was ordered and the specific technology used in the genetic testing process will also influence the type of genetic information generated, having downstream implications for issues of access and use within an EMR.

##### 1. Testing to diagnose and improve treatment

Some genetic tests are used to assist with the diagnosis of a medical condition in a patient who is symptomatic, with the goal of establishing a diagnosis, clarifying prognosis, and influencing medical management. Often, diagnostic tests are offered for Mendelian genetic conditions with childhood onset, and as such usually entail molecular testing of a single gene or a panel of genes that are associated with a small set of related conditions, or in some cases a molecular cytogenetic test (e.g. comparative genomic hybridization, CGH). These conditions make up the majority of the genetic tests that are currently available, and it is important to note that genetic diagnoses can often be made from clinical features (particularly when no genetic test is currently clinically available), biochemical measurements, or DNA based genetic testing. Genetic tests can also be ordered to rule out a potential genetic condition; if the test result is negative this reduces the chances that the patient will have specific symptoms, provides additional information for treatment plans, and has implications for the patient's family.

When contemplating access issues for *diagnostic genetic tests*, it is generally agreed that widespread access to most genetic test results in this category is valuable. First, the genetic test will likely rule in or out a specific differential diagnosis, which has important implications for coordinating care across the medical team. Second, when a condition is genetic, there is an increased likelihood that other family members are affected or are at risk for having affected offspring. When seeing a healthcare provider, medical records to document the condition and specific diagnosis are certainly helpful, but the information about a relative's genetic test results is especially critical in ordering the appropriate test (usually a single-site mutation analysis) and for more clear-cut interpretation of results for these relatives. For example, if a family member is known to have a specific mutation that is not on most standard testing panels this information will allow a healthcare provider to order the proper test on a (yet) unaffected relative and avoid a false negative result. Having these genetic test results available can cut costs for other family members and potentially for insurance providers and the medical center, as testing can be mutation-specific versus a more general testing approach (e.g. sequencing an entire gene or genes). Results may also be available more rapidly. Clear documentation of the specific gene and mutation site in medical records and family letters can allow relatives and their health care professionals to obtain proper testing and maximize test sensitivity and specificity. As such, if the result is provided in a protected part of the medical record or is not easily accessible it may be more difficult for clinicians to identify the relevant information.

Beyond specific diagnostic genetic testing, *pharmacogenetic testing *increasingly can be done to choose a medication that will perform differently in patients with different genetic markers; medical recommendations may be based on pharmacogenetic test results with the hopes of directly improving patient care. For example, genetic variants alter the metabolism of warfarin, and although the evidence base remains under development, testing can improve dosing and make use of the medication safer [19]. Prescribing recommendations, including FDA labeling in some cases [20], are currently available based on pharmacogenomic results including warfarin (for the biomarkers CYP2D9 and VKORC1) and some cancer treatments that are prescribed to individuals on the basis of specific genetic test findings (e.g. Tamoxifen based on Estrogen receptor biomarker results and trastuzumab based on Her2/neu biomarker results). The patient's genetic information is of high relevance to providers when prescribing or reviewing these medications. Having the results of pharmacogenetic tests available in the medical record to all providers, ideally along with "point of care reminders" to offer specific genetic tests before a medication is prescribed and/or regarding appropriate management on the basis of the results, can prevent the patient from receiving ineffective or even harmful medications [21].

In sum, the characteristics of the health conditions that can be tested for with this type of genetic test (they have clear implications for diagnosing and guiding treatment) should likely be the key determining factor for setting access controls on the results. Access controls should be minimized for this type of test, but ideally display of test results should be paired with decision support regarding interpretation of the findings and implications for clinical decisions. Access to genetic information relevant to medication response could still be restricted to clinicians such as clinical pharmacists or available with pharmacy decision support systems rather than automatically to all clinicians.

##### 2. Testing to assist in reproductive choice

Three types of genetic tests assist with reproductive choices: carrier screening testing, pre-implantation genetic testing, and prenatal testing. One thing that these tests have in common is that they do not have direct health implications for the patient, but rather may impact the health of their future children or the health of other relatives. The key determining factor for setting access controls to this type of test should therefore be patient preferences, with default access controls in an EMR more narrowly restricted to reproductive health specialists (e.g. obstetricians) and geneticists.

*Genetic carrier screening *is typically offered when a person is of child-bearing age to predict risks for autosomal recessive (or X-linked) conditions for their offspring. Carrier screening may be population-wide, on the basis of ethnicity, or done on the basis of family history. This type of genetic testing is not medically necessary for the patient, as its primary use is to provide information relevant for reproductive choice and informed decision making. Examples of conditions for which carrier screening is typically done on a population basis and/or offered on the basis of ethnicity include cystic fibrosis and spinal muscular atrophy for women of all ethnic backgrounds; Tay Sachs, Canavan disease and Niemann-Pick disease testing for those of Ashkenazi Jewish heritage; and hemoglobinopathies, such as sickle cell disease, for those with African American backgrounds.

*Preimplantation genetic diagnosis or screening *may be offered for several reasons: as part of in vitro fertilization, when a couple has had difficulty conceiving or maintaining a pregnancy, or as a means of "preconception diagnosis" for an inherited condition for which the potential fetus is at risk. It may also be used for selecting human leukocyte antigen matched children who may be future donors for other offspring who are affected by a serious condition, for gender selection for medical conditions (e.g. an × linked condition carried by the mother), or other personal and potentially controversial reasons.

*Prenatal genetic screening or diagnostic testing *is offered to all pregnant women in the United States to help detect certain conditions during the pregnancy. These genetic tests may be done through analysis of maternal serum analytes (augmented in some cases by ultrasound screening) or by diagnostic genetic analysis of fetal DNA obtained through chorionic villus sampling or amniocentesis. In the near future, it may also become possible to broadly offer noninvasive diagnostic cell free fetal DNA testing through maternal serum [22]. Test results allow the couple to decide whether to continue a pregnancy, or allow the couple to prepare for delivery and childcare or adoption of an affected child.

A major characteristic of genetic testing results for reproductive purposes is that the individual about whom the information reveals personal (and in many cases predictive) genetic health information is not the patient, but rather the offspring. This characteristic is relevant to setting access controls on test results because it raises the question of where the information should be stored. While results may have relevance to the specific pregnancy (or future pregnancies, in the case of accurate recurrence risk counseling), it will not impact the health of the patient in all but rare cases, and misinterpretation of carrier status may result in inappropriate medical care and/or discrimination as was seen, for example, with sickle cell carriers in the 1970s. Several options could be considered to encourage appropriate use of this information in EMRs, including having reproductive genetic test results accessible only to certain care providers (e.g. obstetricians or with patient permission to release, for example, to family members) and automating transfer of relevant records (such as positive test results) to the record of offspring to whom they apply so that key information is then available to care for the individual in the future.

##### 3. Predictive genetic testing

Predictive genetic testing may be done for late-onset conditions for which a patient is not yet symptomatic. These tests can help the patient prepare for managing their medical care and allow him or her to make informed life decisions. Examples of conditions for which predictive testing may be done are Huntington's disease and certain other neurodegenerative diseases (e.g. dementias), as well as Breast and Ovarian Cancer syndrome and certain other cancer syndromes in which a higher risk can be predicted based on genetic test results. Many of these conditions can be caused by mutations inherited from a single parent, cause a high risk (60-100%) of developing the condition (i.e. are high penetrance), and are believed to occur primarily in people with a family history of the condition. But there is increasing variability in this area as genome-wide single nucleotide polymorphism (SNP) genotyping and sequencing technologies improve for more common conditions involving multiple genes (e.g. diabetes, heart disease, and cancers). These conditions are less likely to appear inherited by patients, in part because they are more common in the population and because the various at-risk genotypes convey a far lower risk of developing the condition.

There are two critical issues that impact policies on access to predictive genetic information. The first is that the individual does not yet (and may or may not ever) have the specific condition for which testing is being performed. This opens up the potential for genetic discrimination on the basis of awareness of these test results, and this type of testing is the one for which privacy issues are most salient. In the past some patients have even considered anonymous testing or avoided testing at all to minimize such risks. Again, the current GINA legislation and supplemental state laws provide various levels of protection against health insurance and employment discrimination, but there are still concerns that suggest that some level of protected access would be most appropriate, either by patient consent or to specific health care providers depending on the exact genetic test result. The second issue that arises with predictive genetic test results is difficulty with interpretation. Clinician training is therefore critical for setting policies on access to this type of genetic test results. Many healthcare workers are unfamiliar with the concept of decreased penetrance, and may inappropriately tell patients that these test results are fully predictive. Decision support tools that educate about the role of the environment (where appropriate) and early screening may help improve patient education and outcomes.

#### B. Interpreting genetic test results and the need for educational prompts in the EMR

Regardless of the reason for ordering a genetic test, the test result itself can give the health care provider different types of information and have varying levels of interpretability. While analytic validity is usually high, there is much less consistency in the degree of clinical validity and clinical utility of genetic testing at this time (the clinical impact of a genetic mutation and whether there are clinical interventions to be made based on the result) [23]. Additionally, genetic test development is not yet regulated by the Clinical Laboratory Improvement Amendments (CLIA) oversight process. There is tremendous variability in the degree of information and interpretation of results returned by different laboratories, making it challenging for a non-genetics specialist to interpret genetic test results accurately [24,25]. These issues can create significant ambiguity about result interpretation, and decisions regarding medical record access to these results should consider the clinical utility of the findings and the availability of clinical decision support systems to guide healthcare provider use of test findings. Results without clear clinical utility should either be restricted to genetic specialists for interpretation, or at a minimum not be prominently displayed in EMR interfaces.

##### 1. Simple, certain test results

An example of a straightforward result to interpret would be from a genetic test for a Mendelian disorder for which (a) there is a single gene locus affected, and/or a single mutation that occurs for the condition, and (b) high likelihood the disorder will express itself when the mutation is present. These test results would show that the mutation is either present or not, and give the patient a very clear result with a clear outcome. Few genetic tests fit neatly into this category. One example is Huntington's disease (HD), which is caused by a genetic mutation where a specific trinucleotide sequence is repeated a variable number of times, and persons with a number of these repeated trinucleotides over a certain threshold will develop HD. A result from a test for HD would typically provide the number of expanded repeats as well as the normal and abnormal ranges for the result; a positive result (e.g. 52 repeats on one allele) means that the patient does have HD or will develop it if they live long enough, and a negative result (both alleles less than 30) means that the patient does not and will not have HD. Despite this apparently clear test result, there remains variability in the age of onset and the progression of symptoms that cannot be definitively predicted on the basis of the genetic test, and there are other diseases which mimic the symptoms of HD.

Another relatively straightforward test result would be a prenatal chromosome analysis from an amniocentesis, called a karyotype, in which all of the cells analyzed had the same unambiguous chromosome count with no extra or missing pieces of chromosomes and no chromosome inversions or translocations. The certainty of the test result, such as a positive result for Down syndrome in which an extra chromosome 21 is seen, would be very high (over 99.8%). Depending on the lab, however, the report with this karyotype result may or may not state the reason for the test (e.g. elevated risk for Down syndrome), interpret the results as normal or otherwise, include an image of the chromosomes, or state the gender. Given the certainty of this type of test results, providing broad clinical access to findings in combination with clear decision support and/or provider education is encouraged.

##### 2. Complex test results: analytically valid results with clinical uncertainty

A genetic test may have very certain results and high analytic validity but the result may not be simple to interpret and therefore have unclear clinical validity or utility that will likely change over time as technologies and our clinical knowledge improve. When test results have unclear clinical validity or utility it may be appropriate for access to the results to be automatically restricted to genetic specialists unless there is additional intervention. Moreover, decision support prompts should be added to the EMR to provide educational information about the clinical uncertainty that is present, and suggest additional consultations with experts. For example, one possible result of a karyotype or molecular cytogenetic analysis (a test which looks for specific genetic sequences on chromosomes) is that the same genetic complement is seen in every cell tested, but the complement contains complicated duplications and deletions of parts of chromosomes (copy number variants or CNVs) rather than the normal amount of genetic information. While it is highly certain that this result is accurate, it is complicated to interpret and the formulation of a prognosis may be difficult. A clinician interpreting a test result that identifies CNVs must review the literature for case reports and additional information about the specific duplications or deletions, assessing potentially affected genes and their functions. All or part of this information may not be readily available, it may change over time, and a lab may not report all of this information along with the results. Additional testing is often required to further clarify the interpretation (e.g. on parents, to determine if the CNV was inherited or de novo).

Testing complexity also arises when a gene has many potentially detectable mutations (high allelic heterogeneity) and various testing approaches can be taken, influencing the potential sensitivity of the test performed. An important though not unique example of this situation is Cystic Fibrosis (CF) screening [26]. CF is caused by mutations in a single gene (*CFTR*); however more than 1500 different changes in this one gene can cause CF. Because a patient must carry a mutation on both copies of their *CFTR *gene to have CF they frequently have two different mutations (compound heterozygosity), making prognostication difficult since genotype-phenotype correlations are limited. Modifier genes, most of which are unknown at this time, can result in patients with the same combination of mutations showing different symptoms, even within the same family, making it even more difficult to prognosticate.

There are also several CF testing methods available [26]: targeted mutation panels which test for a limited number of the more common mutations and full gene sequencing which would detect a higher percentage of mutations across the gene but may not detect large duplications or deletions or some types of peripheral mutations. The testing method affects the test sensitivity, making the results more or less certain as well as more challenging to accurately interpret. A typical CF panel test report contains a list in genetic notation of all of the mutations that were tested for and whether any were found. The interpretation of the report is typically left to the provider who receives the report from the lab. Because the test sensitivity is not 100%, a negative result (e.g. a result where an asymptomatic person is found to carry no identifiable mutation) means that there are no mutations which could be found by the selected test panel, but it does not mean there are no mutations present. There is always some residual risk that a disease-causing mutation was not detected by the test, in part because the test panel does not cover all possible mutations. Misinterpretation of a critical point like this could lead a patient to falsely believe that they are not at risk of having an affected child and potentially create malpractice issues. Additionally, testing panels can change over time making it complicated to know if and when previously negative testing should be repeated. Point of service reminders, educational prompts, and recommendations to consult specialists would all be useful decision support tools within an EMR to help providers maintain awareness of the changing pace of genetic technologies.

Finally, tests for single nucleotide variants (SNVs) are the types of tests most commonly done by direct-to-consumer genetic testing companies and during research studies in an attempt to find genome-wide associations for certain diseases or conditions. Testing methods for SNVs have high analytic validity and can reliably detect whether a SNV that was found to be associated with disease risk is present or not. However, the clinical validity of such testing, in other words the association between a certain SNV and having a higher risk for a certain disease, is usually fairly weak. This leaves the provider with significant uncertainty about the implications for the patient. For example, not all persons with a specific SNV will develop colorectal cancer, and persons without the variant may develop the cancer anyway. In addition, some conditions may have many SNVs associated with them, and there are not yet robust methods for combining and weighing the risks across an array of SNVs. Finally, several studies have shown significant variation in the interpretation of absolute risk assigned by the various testing companies even on the basis of the same genotype information [27,28], and the ultimate clinical interpretation can vary as new SNVs are uncovered or as their usage varies across companies [29]. Given the generally weak clinical validity of these genetic test results, a policy that defaults to restricting access to them to genetic specialists may prevent unwarranted clinical concern or misguided treatments based on misunderstanding the results. As methods for better estimating risk are generated, decision support systems will be required to effectively use these algorithms.

## Summary

Electronic medical records enhance both the ability of a health care system to store complex, lengthy genetic test result data in a patient's record and the ability of the system to control access to that informa-tion. Test results placed in an EMR can be more easily accessed by a wider range of providers who do not have to request and wait for a paper chart, and also offer an opportunity to build in privacy safeguards which cannot be built into a paper chart. For example, EMRs often allow access to psychiatric records to be more restricted than access to cholesterol test results, and they can allow prenatal providers to flag a record when a patient does not wish (for example) to know the gender of her fetus. Similarly, a health care system could place additional privacy restrictions around genetic information pertaining to sensitive conditions, such as adult onset disorders like Huntington's disease or cancer susceptibility genes. The type and extent of protection, if any, is currently left up to the institution or department to decide upon and implement, therefore there is little consistency between institutions in the use of these privacy safeguards in EMRs [3]. (This extra privacy is typically created because of the sensitivity of the information not because it is genetic in nature).

Genetic testing can provide clear benefits to patients and providers in terms of improving diagnosis, prognostication, and treatment planning. However, these tests also pose risks to patients of stigma-tization, distress, and dissatisfaction, and risks to providers of misinterpretation and subsequent errors in treatment recommendations. There are also inevitable risks of miscommunication between patients and providers and between different providers with access to test results. EMRs have the potential to provide clinicians with better access to genetic test results, but without specific forethought in the design of these systems this may not necessarily lead to better interpretation or clinical care. Health care systems must carefully consider when access to genetic test results and decision support for their interpretation is safe and desirable and how to implement systems to prevent sharing and access when the risks are unacceptable. We believe there are three key dimensions to these access decisions: (1) the specific characteristics of the test and the resulting raw data, including sensitivity, interpretability, possible secondary findings, and the likely sensitivity of results for patients, (2) patient preferences for access and use of genetic information in their health care, and (3) level of clinician training in genomic medicine, test interpretation, and patient counseling. Decisions about the extent of clinician access to test results should vary based on these three factors in ways we have suggested above.

Genetic tests have widely varying implications for patients' immediate and future health care decisions, as well as for their family members. For optimal use and patient acceptance of genetic testing as part of medical care, systems and policies should ensure appropriate use of and access to genetic information [30]. There is no *a priori *reason for EMRs to treat all genetic test results the same, with uniform policies on restricting or allowing access. We recommend implementing some level of access control on sensitive genetic (and other) data, specifically controls around data access for non-specialist providers and controls that consider patients' input into allowed uses of genetic test results they consider sensitive. Providers trained in genetics, including clinical geneticists and genetic counselors, should be involved in the specification of these record systems as they are best trained to interpret and understand the proper use of results from the diverse types of genetic tests and predict the impacts of testing and results on patients with diverse conditions. We encourage EMR developers to consider building in point-of-care reminders or decision support tools [31] around genetic data and screening recommendations to support health care providers in remaining up-to-date in the implications of genetic test results for medical management. Finally, we also strongly urge health care systems to be cautious managing multiplex genetic test results that provide information beyond a single specific purpose, as the patient may not have consented to or considered the implications of all the test results.

Health IT systems have the potential to greatly facilitate using genetic information to both improve health outcomes and respect the individual preferences of patients regarding their care. To facilitate this work we suggest that any panels creating clinical guidelines for the use of genetic test results in clinical care include decision support and health information technology experts to ensure recommendations can be translated into tools that adequately guide practice. As more and more health care systems implement genetic testing in the context of comprehensive electronic medical records, balancing clinical possibilities and patient preferences will be a significant challenge to their successful implementation and crucial to patient acceptance and clinical adoption of the potential treatment advances from genomic medicine.

## Abbreviations

ALS: Amyotrophic Lateral Sclerosis; BRCA1, BRCA2: BReast CAncer genes; CF: Cystic Fibrosis; CNV: Copy number variants; DNA: deoxyribonucleic acid; EMR: electronic medical record; GINA: Genetic Insurance Nondiscrimination Act; HD: Huntington's disease; SNP: single nucleotide polymorphism; SNV: single nucleotide variant.

## Competing interests

The authors declare that they have no competing interests.

## Authors' contributions

Each of the authors contributed to the paper as follows: JAT, ETL, and KEO conceived of the study, and participated in its design, coordination, and revisions of the manuscript. DCD outlined the structure of the paper, reviewed literature, and drafted the manuscript, with contributions from all authors including DJC. All authors read and approved the final manuscript.

## Author's information

DCD is a board certified genetic counselor, has a master's degree in human genetics and genetic counseling, and currently works for Santa Clara Valley Medical Center in San Jose, California, and Palo Alto Medical Foundation in Mountain View, California.

ETL has a PhD in Organizational Sociology and is a health services researcher at the Center for Health Care Evaluation in the US Department of Veterans Affairs.

KEO is a board certified genetic counselor and an Associate Professor in the Department of Genetics. She serves as the Program Director for the MS in Human Genetics and Genetic Counseling at Stanford University.

DJC has an MD and Ph.D. in Pharmacology, is a practicing anesthesiologist at the VA Palo Alto Health Care System, and an Associate Professor of Anesthesiology at Stanford University.

JAT has a PhD in Neurobiology, and is an investigator at the Center for Health Care Evaluation in the US Department of Veterans Affairs and Director of the VA Program Evaluation and Resource Center.

## Pre-publication history

The pre-publication history for this paper can be accessed here:

http://www.biomedcentral.com/1472-6963/11/294/prepub
